# Effect of Compound Danshen Dripping Pills on cardiac function after acute anterior ST-segment elevation myocardial infarction: A randomized trial

**DOI:** 10.7555/JBR.38.20240325

**Published:** 2025-01-10

**Authors:** Bo Deng, Sibo Wang, Yujie Wu, Qiming Wang, Rui Qiao, Xiwen Zhang, Yuan Lu, Li Wang, Shunzhong Gu, Yuqing Zhang, Kaiqiao Li, Zongliang Yu, Lixing Wu, Shengbiao Zhao, Shuanglin Zhou, Yang Yang, Liansheng Wang

**Affiliations:** 1 Department of Cardiology, the First Affiliated Hospital of Nanjing Medical University, Nanjing, Jiangsu 210029, China; 2 Department of Cardiology, Anqing Municipal Hospital Affiliated to Anhui Medical University, Anqing, Anhui 246003, China; 3 Department of Cardiology, Huai'an First People's Hospital, Huai'an, Jiangsu 223300, China; 4 Department of Cardiology, the Affiliated Hospital of Xuzhou Medical University, Xuzhou, Jiangsu 221006, China; 5 Department of Cardiology, Pukou Branch of Jiangsu People's Hospital, Nanjing, Jiangsu 211899, China; 6 Department of Cardiology, Hai'an People's Hospital, Nantong, Jiangsu 226699, China; 7 Department of Cardiology, the Affiliated Jiangning Hospital of Nanjing Medical University, Nanjing, Jiangsu 211199, China; 8 Department of Cardiology, Qixia District Hospital of Nanjing City, Nanjing, Jiangsu 210046, China; 9 Department of Cardiology, the First People's Hospital of Kunshan Affiliated to Jiangsu University, Kunshan, Jiangsu 215300, China; 10 Department of Cardiology, Nanjing Lishui District Hospital of Traditional Chinese Medicine, Nanjing, Jiangsu 211299, China; 11 Department of Cardiology, Nanjing Meishan Hospital, Nanjing, Jiangsu 210039, China

**Keywords:** Compound Danshen Dripping Pills, cardiac function, acute anterior myocardial infarction, randomized controlled trial

## Abstract

The current study aimed to evaluate the efficacy and safety of Compound Danshen Dripping Pills (CDDP) in improving cardiac function in patients with acute anterior ST-segment elevation myocardial infarction (AAMI). Between February 2021 and February 2023, 247 eligible patients with AAMI after primary percutaneous coronary intervention were enrolled and randomly assigned (1∶1) to receive CDDP (*n* = 126) or placebo (*n* = 121), with a follow-up of 48 weeks. Compared with the placebo group, the CDDP group demonstrated a significant increase in left ventricular ejection fraction values after 24 weeks of treatment (least squares mean: 3.31; 95% confidence interval [CI]: 1.72–4.90; *P* < 0.001) and at the 48-week follow-up (least squares mean: 4.35; 95% CI: 2.76–5.94; *P* < 0.001). Significant reductions in N-terminal pro-B-type natriuretic peptide levels were observed in both groups at the 24- and 48-week visits with no significant difference between the two groups (*P* > 0.1 for all). The incidence of major adverse cardiovascular and cerebrovascular events was 6.35% in the CDDP group and 5.79% in the placebo group (*P* = 0.822). Notably, no serious adverse events were attributed to CDDP. These findings suggest that CDDP may be well tolerated and could improve left ventricular ejection fraction in patients with AAMI at 24 and 48 weeks.

## Introduction

Adverse ventricular remodeling post-acute myocardial infarction (AMI) is a common pathological change in cardiac structure and function that usually involves various regulatory factors, such as mechanical, neurohormonal, cardiac inflammatory and immune environments, ischemia/reperfusion injury, energy metabolism, and genetic factors^[1–3]^. Thus, AMI is a significant contributor to heart failure (HF) and reduces patient survival^[4]^. Although primary percutaneous coronary intervention (PPCI), as an early revascularization strategy, combined with cardioprotective drugs, may significantly improve myocardial perfusion, the risk of HF after myocardial infarction has not been eliminated. Therefore, treatment for ventricular remodeling requires further optimization.

Therapies targeting neurohormonal activation, such as beta-blockers and renin-angiotensin-aldosterone system inhibitors, have improved outcomes in AMI. In preclinical studies, the ATP-sensitive potassium channel opener nicorandil and calcium channel blockers have also shown potential to improve ventricular remodeling^[5–6]^. However, these patients remain at an elevated risk^[7]^. Compound Danshen Dripping Pill (CDDP), a traditional Chinese medicine preparation composed of *Salvia miltiorrhiza* Bunge, *Panax notoginseng* (Burkill), and borneol, has gradually gained researchers' attention because of its unique medical value in the treatment of cardiovascular disease. Some studies have shown that CDDP may not only dilate coronary vessels, increase coronary blood flow, and inhibit platelet aggregation, but also regulate blood lipids^[8]^, prevent atherosclerosis, and protect the vascular endothelium^[9]^. In addition, CDDP may treat microcirculation disorders caused by ischemia-reperfusion injury^[10]^, improve energy metabolism after myocardial infarction, and contribute to the conversion to normal glucose metabolism^[11]^, which in turn reduces the risk of recurrent angina pectoris, no-reflow phenomena, and HF. All of these indicate that CDDP may have a protective effect in the treatment of myocardial infarction^[12]^. However, studies on the treatment of acute anterior ST-segment elevation myocardial infarction (AAMI) with CDDP remain limited.

To date, few studies have investigated the safety and efficacy of CDDP in clinical applications for AAMI patients. In the current study, we conducted a multicenter, exploratory, randomized controlled trial, using echocardiography to observe the changes in ventricular dimensions and cardiac output to assess the effects of CDDP on ventricular remodeling and cardiac function post-AAMI.

## Materials and methods

### Ethics and trial registration

The CODE-AAMI trial was a multicenter, randomized, double-blind, placebo-controlled clinical trial. The trial design has been previously detailed in the protocol^[13]^. Patients with AAMI undergoing percutaneous coronary intervention (PCI) were recruited from 11 clinical centers across various regions of China between February 2021 and February 2023, with a follow-up of 48 weeks until January 2024.

Ethical approval for the current study (No. 2019-SR-204) was obtained from the Independent Ethics Committee of the First Affiliated Hospital of Nanjing Medical University and other participating centers. The study was conducted in accordance with Good Clinical Practice and the Declaration of Helsinki, and was registered at https://clinicaltrials.gov/ (Identifier: NCT0500041) on August 28, 2021. A written informed consent form was obtained from each participant before study initiation.

### Inclusion and exclusion criteria

The inclusion criteria were as follows: (1) aged 18–75 years, with no sex restrictions; (2) documented diagnosis of AAMI according to the guidelines for the diagnosis and treatment of acute ST-segment elevation myocardial infarction (2019)^[14]^; (3) patients with primary AMI; (4) patients who completed PCI reperfusion within 12 h of symptom onset; (5) Killip class Ⅰ–Ⅲ^[15]^; and (6) subjects who participated voluntarily and signed the informed consent.

The exclusion criteria were as follows: (1) cardiac insufficiency caused by other diseases (*e.g.*, valvular heart disease, congenital heart disease, pericardial disease, arrhythmias, and other non-cardiac causes); (2) coronary artery bypass graft surgery within the past 12 weeks; (3) currently undergoing cardiac resynchronization therapy; (4) left ventricular outflow tract obstruction; (5) myocarditis; (6) uncontrolled severe arrhythmia; (7) aortic aneurysm; (8) severe liver, kidney, hematologic, psychiatric, or systemic diseases; (9) significant liver or kidney dysfunction (serum alanine aminotransferase [ALT] > 2.0 times the upper limit of normal; serum creatinine > 1.5 times the upper limit of normal); (10) serum potassium > 5.5 mmol/L; (11) uncontrolled hypertension (> 180/110 mmHg); (12) pregnant or lactating individuals; (13) allergy to CDDP; and (14) participation in other drug clinical trials.

### Randomization, blinding, and interventions

Eligible patients were randomly assigned in a 1∶1 ratio to receive either placebo (containing no CDDP but ingredients identical to the CDDP group, and produced by Tasly Pharmaceutical Group Co., Ltd., Tianjin, China) or CDDP in addition to routine treatment. Random allocation was generated automatically using a stratified technique by a centralized web-based tool (http://www.cnrres.co.kr/valid). Blinding was strictly maintained for both the investigators and the participants throughout the trial. All study drugs underwent quality inspection. Subjects in the CDDP group received a 24-week treatment course, consisting of 20 tablets of CDDP administered before PPCI and 10 tablets three times daily after PPCI, while the control group received a placebo at the same intervals. Basic treatment for AAMI remained unchanged, and patients maintained a regular lifestyle, including an appropriate diet and rest.

### Primary outcomes

Echocardiography was performed at seven days, four weeks ± seven days, 24 weeks ± 15 days, and 48 weeks ± 15 days post-PPCI. The primary outcome of the study was the change in the echocardiographic indices of left ventricular (LV) remodeling, as measured by two-dimensional echocardiography. These indices included left ventricular ejection fraction (LVEF), defined as the percentage of stroke volume to LV end-diastolic volume, and LV dimensions at end-systole and end-diastole (LVDs and LVDd) during the study period. The occurrence of malignant arrhythmias post-myocardial infarction, including frequent ventricular premature beats, ventricular tachycardia, ventricular flutter, or ventricular fibrillation, was managed through antiarrhythmic agents, electrical defibrillation, or cardioversion during echocardiography. A widely used ultrasound system (Philips Medical Systems, MA, USA) was employed to measure LV diameter and ejection fraction. Echocardiographic records from the participating institutions were sent to a central core laboratory for analysis.

### Secondary outcomes

Secondary outcomes included the decline in N-terminal pro-B-type natriuretic peptide (NT-proBNP) levels, as measured *via* blood testing, adverse cardiovascular events (including death, cardiac arrest, or cardiopulmonary resuscitation, readmission because of HF or angina pectoris, worsening cardiac function, and stroke), and the occurrence of arrhythmias (assessed by dynamic electrocardiogram). Safety indicators included (1) vital signs, such as changes in body temperature, heart rate, respiration, and blood pressure, at each visit; (2) laboratory tests, including complete blood count, liver and kidney function, serum electrolytes, and lipoproteins; (3) treatment-related adverse events graded according to the common terminology criteria for adverse events (CTCAE) version 4.0^[16]^, with a severity scale ranging from one (mild) to five (death); and (4) Holter monitoring. NT-proBNP levels were measured at baseline, three days, seven days, four weeks ± seven days, 24 weeks ± 15 days, and 48 weeks ± 15 days post-PPCI. Holter monitoring was conducted seven days post-PPCI.

### Sample size estimation

Based on relevant research results^[10,17–18]^, the coefficient of variation of LVEF levels in the control group was assumed to be 1.5. The number of participants in the trial and control groups was set at a 1∶1 ratio, with α set at 0.05 (two-sided) and statistical power at 90%. Considering a dropout rate of no more than 10%, the required sample size was 134 participants per group, for a total of 268 participants. Finally, we enrolled 247 participants for the trial, with 126 in the CDDP group and 121 in the placebo group. Considering the relatively high dropout rate, we recalculated the statistical power using the actual sample of 247 subjects, and the actual statistical power in this trial was 83%, which is still acceptable.

### Statistical analysis

Baseline characteristics were presented as mean ± standard deviation (SD) or median and interquartile range for continuous variables and as counts and percentages (%) for categorical variables. The analysis was performed according to the intent-to-treat principle and included all patients who received the randomized medication and completed baseline evaluation, with at least one post-baseline follow-up visit. For the efficacy analysis population, participants were included in the analysis according to their randomized allocation. Missing outcome data were handled through multiple-imputation analysis, which incorporated all baseline characteristics (age, sex, body mass index, blood pressure, medical history, and medications), outcomes at all time points, and the treatment indicators to generate five imputed datasets. The outcomes of multiple-imputation analysis were compared with those of complete-case analysis, which served as a sensitivity analysis.

Comparisons of changes in primary endpoints (LVEF, LVDd, and LVDs) and NT-proBNP from the baseline were performed between the two groups, with *P* < 0.05 (two-tailed) considered statistically significant, using a mixed-effect model for repeated measures. Least squares means and 95% confidence intervals (CIs) were calculated using a linear mixed-effect model for repeated measures, with group, baseline values, time of evaluation, and the interaction term between treatment group and time of evaluation as fixed effects. Patient-specific effects were entered into the model as random effects, assumed to follow a normal distribution with a mean value of zero. Other exploratory outcomes (ALT, total cholesterol, triglycerides, blood urea nitrogen, serum creatinine, uric acid, and potassium) were also evaluated using the same method. Some parameters, such as LVDs and NT-proBNP, were natural log-transformed and analyzed within the model. For adverse events, the number of patients was calculated and compared between the two groups using the Chi-square or Fisher's exact test when appropriate. Statistical analysis was performed using SPSS version 25.0 (SPSS, Inc., Armonk, NY, USA).

## Results

### Patients

**Figure 1:**
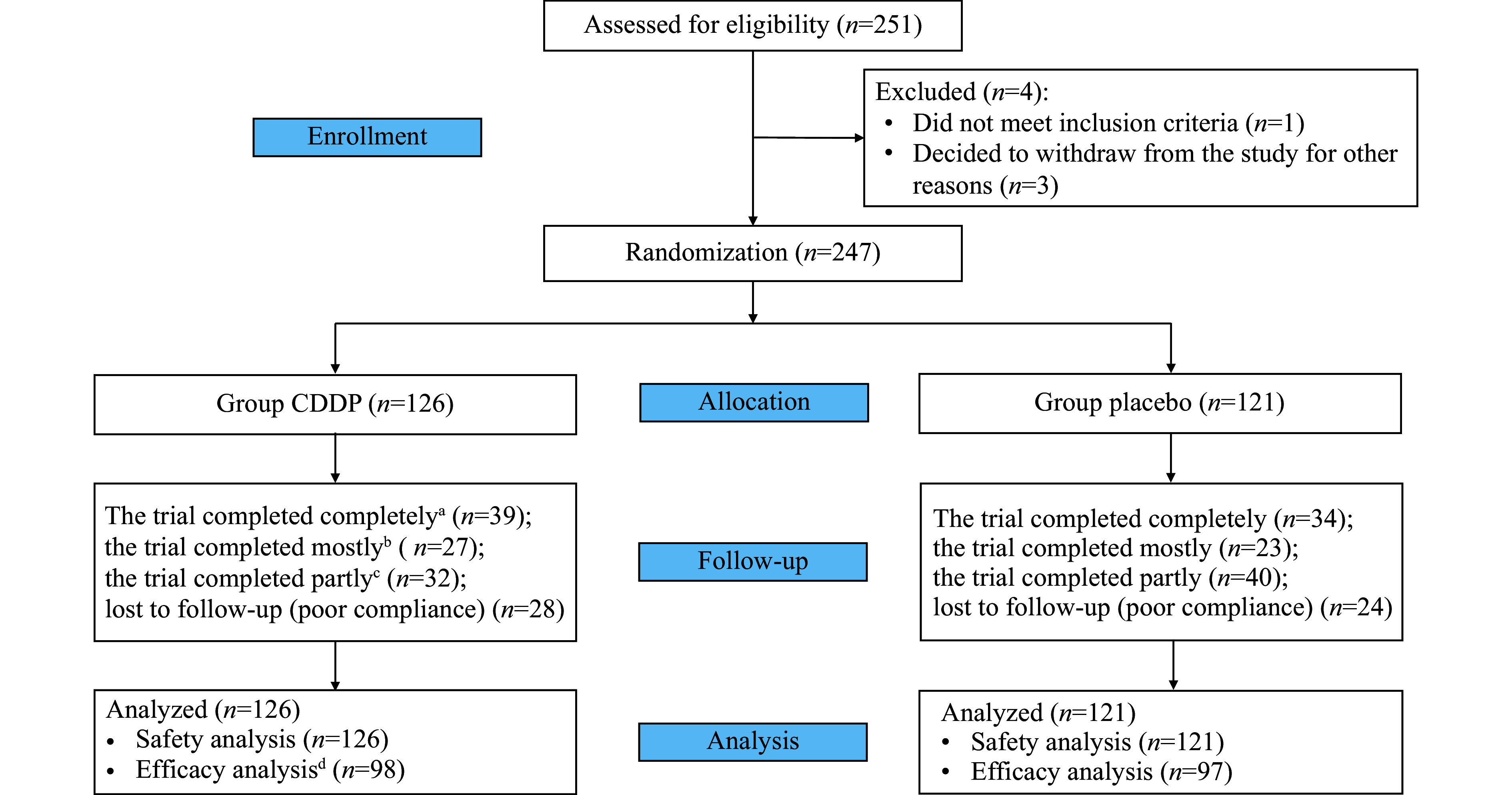
Flow chart of participant enrollment in the CODE-AAMI trial. ^a^Three of the four-week, 24-week, or 48-week follow-up visits were completed; ^b^Two of the three visits were completed; ^c^One of the three visits was completed. ^d^Participants who underwent at least one follow-up measurement were included in the efficacy analysis.

Between February 21, 2021, and February 12, 2023, a total of 251 patients were screened for this study, of whom 247 across 11 clinical centers underwent randomization: 126 were assigned to the CDDP group and 121 to the placebo group (***Fig. 1***), all of whom took the study drugs until their last hospital visit according to case report forms written by investigators from the corresponding clinical centers. During the follow-up period of four weeks, 24 weeks, and 48 weeks after PPCI, a total of 52 patients (28 in the CDDP group and 24 in the placebo group), who did not complete any of the three visits, were excluded from the efficacy analysis population. The follow-up ended on January 29, 2024.

Overall, the mean (SD) age of the patients was 59.06 (11.73) years, the mean (SD) body mass index was 25.00 (3.23), and 205 patients (83.00%) were male. The majority of patients received aspirin, P2Y12 receptor inhibitors, and statins during hospitalization, while nearly two-thirds of patients received β-receptor blockers, and more than half received angiotensin-converting enzyme inhibitors (ACEIs), angiotensin Ⅱ receptor blockers (ARBs), or angiotensin receptor neprilysin inhibitors (ARNIs). The baseline characteristics of the two groups are shown in ***Table 1***.

**Table 1:** Baseline characteristics of patients in both groups

Characteristics	Group CDDP^a^ (*n*=126)	Group placebo^a^ (*n*=121)	Statistics^b^	*P*-value
Age (years, mean±SD)	60.55±11.83	57.52±11.46	*t*=2.043	0.042
Male [*n* (%)]	106 (84.13)	99 (81.82)	*χ*^*2*^=0.233	0.629
Body mass index (kg/m^2^, mean±SD)	24.64±3.25	25.38±3.18	*t*=−1.808	0.073
Coexisting diseases [*n* (%)]				
Hypertension	69 (54.76)	51 (42.15)	*χ*^*2*^=3.931	0.047
Diabetes	32 (25.40)	33 (27.27)	*χ*^*2*^=0.112	0.738
Hyperlipidemia	18 (14.29)	22 (18.18)	*χ*^*2*^=0.690	0.406
Cerebrovascular diseases	13 (10.32)	8 (6.61)	*χ*^*2*^=1.090	0.297
Current smoking [*n* (%)]	60 (47.62)	60 (49.59)	*χ*^*2*^=0.096	0.757
Systolic pressure (mmHg, mean±SD)	129.51±22.21	129.01±21.74	*t*=0.179	0.858
Diastolic pressure (mmHg, mean±SD)	80.75±15.00	81.95±13.80	*t*=−0.654	0.515
Heart rate (beats per min, mean±SD)	81.09±15.29	79.97±15.32	*t*=0.575	0.566
Concomitant medications [*n* (%)]				
Aspirin	105 (83.33)	108 (89.26)	*χ*^*2*^=1.824	0.177
P2Y12 receptor inhibitors	115 (91.27)	117 (96.69)	*χ*^*2*^=3.184	0.074
Statins	110 (87.30)	110 (90.91)	*χ*^*2*^=0.825	0.364
Ezetimibe	31 (24.60)	34 (28.10)	*χ*^*2*^=0.389	0.533
Beta-receptor blockers	74 (58.73)	83 (68.60)	*χ*^*2*^=2.594	0.107
ACEIs/ARBs/ARNIs	73 (57.94)	72 (59.50)	*χ*^*2*^=0.063	0.802
SGLT-2 inhibitors	21 (16.67)	26 (21.49)	*χ*^*2*^=0.931	0.335
MRA	13 (10.32)	19 (15.70)	*χ*^*2*^=1.587	0.208
Loop diuretics	15 (11.90)	17 (14.05)	*χ*^*2*^=0.252	0.616
Nitrates	8 (6.35)	15 (12.40)	*χ*^*2*^=2.673	0.102
Echocardiographic values				
LVEF (%, mean±SD)	52.28±7.05	53.40±6.12	*t*=−1.331	0.184
LVDd (mm, mean±SD)	48.87±4.52	48.94±1.14	*t*=−0.165	0.869
LVDs (mm, median [IQR])	33.26 (31.00, 36.07)	32.44 (30.04, 36.00)	*Z*=−1.488	0.137
NT-proBNP (pg/mL, median [IQR])	825.05 (213.25, 1260.91)	872.96 (388.35, 1388.29)	*Z*=−0.303	0.762
^a^Eligible patients were randomized into the CDDP and placebo groups.^b^Data that conformed to a normal distribution were expressed as the mean ± standard deviation (SD) and analyzed using Student's *t*-tests. Data that conformed to a non-normal distribution were expressed as the median (interquartile range) and analyzed using the Mann-Whitney *U* test. The Chi-square test was used to compare categorical data (sex, coexisting diseases, current smoking, concomitant medications, and LVEF). Abbreviations: CDDP, Compound Danshen Dripping Pills; SD, standard deviation; ACEIs, angiotensin-converting enzyme inhibitors; ARBs, angiotensin Ⅱ receptor blockers; ARNIs, angiotensin receptor neprilysin inhibitors; SGLT-2, sodium-dependent glucose transporters 2; MRA, mineralocorticoid-receptor antagonists; LVEF, left ventricular ejection fraction; LVDd, left ventricular end-diastolic dimension; LVDs, left ventricular end-systolic dimension; IQR, interquartile range; NT-proBNP, N-terminal pro-B-type natriuretic peptide.				

### Primary outcomes

**Figure 2:**
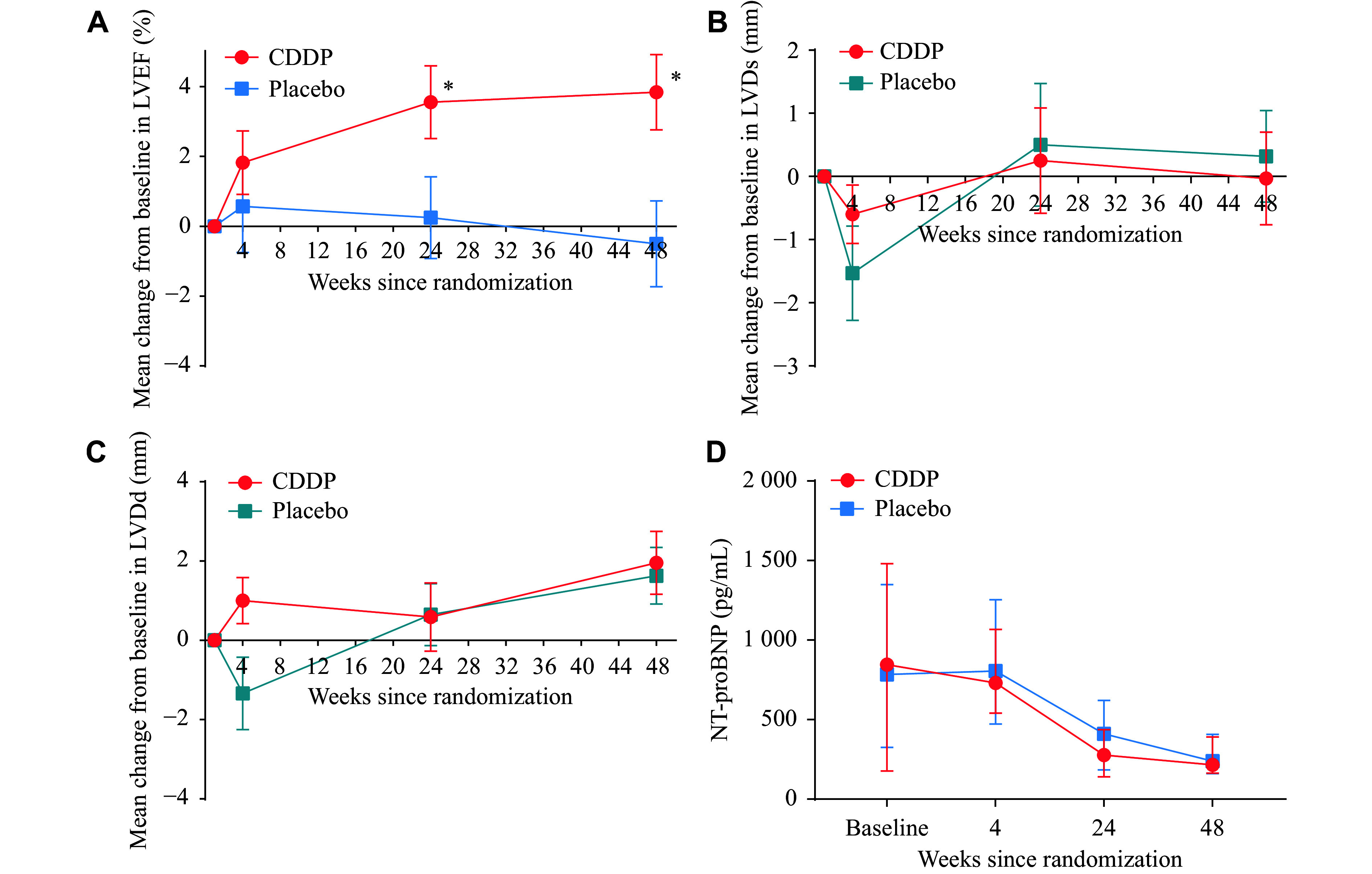
Change in echocardiography measurements and NT-proBNP levels from baseline to four, 24, and 48 weeks after PCI. A: Comparison of the change in LVEF. B: Comparison of the change in LVDs. C: Comparison of the change in LVDd. The differences from each evaluation point to baseline between the two treatment groups were expressed as mean (95% CI) and analyzed by linear mixed effects models for repeated measures (A–C). ^*^P < 0.001. D: Comparison of NT-proBNP levels. NT-proBNP in both groups was expressed as median (IQR) and analyzed by linear mixed effects models for repeated measures. Abbreviations: LVEF, left ventricular ejection fraction; LVDs, left ventricular end-systolic dimension; LVDd, left ventricular end-diastolic dimension; NT-proBNP, N-terminal pro-B-type natriuretic peptide; CI, confidence interval.

At baseline and the following four-, 24-, and 48-week visits, echocardiography tests were performed. As shown in ***Fig. 2*** and ***Table 2***, patients who received CDDP treatment had greater improvements in LVEF, compared with those who received placebo treatment at the 24-week visit (least squares mean: 3.31; 95% CI: 1.72–4.90; *P* < 0.001) and the 48-week visit (least squares mean: 4.35; 95% CI: 2.76–5.94; *P* < 0.001), but not in LVDd or LVDs (all *P* > 0.05 at the 24- and 48-week visits). The result of the complete-case analysis was similar to that of the multiple-imputation analysis (***Supplementary Table 1***, available online).

**Table 2:** Primary, secondary, and exploratory endpoints

Variables^a^	Four weeks			24 weeks			48 weeks	
	Difference (95% CI)^b^	*P*-value		Difference (95% CI)	*P*-value		Difference (95% CI)	*P*-value
LVEF	1.25 (−0.34, 2.84)	0.122		3.31 (1.72, 4.90)	<0.001		4.35 (2.76, 5.94)	<0.001
LVDd	2.33 (1.24, 3.43)	<0.001		−0.06 (−1.15, 1.04)	0.919		0.33 (−0.77, 1.43)	0.554
lgLVDs	0.01 (−0.01, 0.03)	0.082		−0.01 (−0.02, 0.01)	0.366		−0.01 (−0.02, 0.01)	0.294
lgpro-BNP	0.12 (−0.07, 0.31)	0.206		−0.13 (−0.32, 0.06)	0.175		−0.02 (−0.21, 0.17)	0.824
lgALT	−0.02 (−0.13, 0.08)	0.651		0.01 (−0.10, 0.11)	0.912		0.04 (−0.07, 0.14)	0.489
lgTC	0.01 (−0.04, 0.05)	0.709		0.02 (−0.03, 0.06)	0.417		0.04 (−0.01, 0.08)	0.097
lgTG	−0.01 (−0.09, 0.08)	0.924		0.03 (−0.06, 0.12)	0.489		0.01 (−0.07, 0.09)	0.778
lgBUN	−0.01 (−0.07, 0.04)	0.655		−0.03 (−0.08, 0.03)	0.372		−0.02 (−0.08, 0.03)	0.413
lgScr	0.01 (−0.04, 0.05)	0.825		0.01 (−0.04, 0.05)	0.871		−0.01 (−0.05, 0.04)	0.738
UA	−1.35 (−37.01, 34.32)	0.941		−3.24 (−38.91, 32.42)	0.858		−3.36 (−39.02, 32.31)	0.853
K^+^	−0.03 (−0.18, 0.12)	0.727		−0.05 (−0.20, 0.10)	0.481		0.02 (−0.13, 0.16)	0.837
^a^Eligible patients were randomized into the CDDP and placebo groups.^b^The differences from each evaluation point to baseline between the two treatment groups are based on the difference of least square means of linear mixed effects models for repeated measures.Abbreviations: LVEF, left ventricular ejection fraction; LVDd, left ventricular end-diastolic dimension; LVDs, left ventricular end-systolic dimension; NT-proBNP, N-terminal pro-B-type natriuretic peptide; ALT, alanine transaminase; TC, total cholesterol; TG, triglyceride; BUN, blood urea nitrogen; Scr, serum creatinine; UA, uric acid; K, potassium.								

### Secondary outcomes

#### Change in plasma NT-proBNP level

Serum NT-proBNP levels were measured at each visit, and the two groups had similar median NT-proBNP levels at baseline (***Table 1***). Overall, there was a significant reduction in median NT-proBNP levels throughout the treatment period (***Fig. 2D***). However, no significant differences were observed in CDDP treatment effects at the four-, 24-, and 48-week visits, compared with the placebo group (*P* > 0.1 for all; ***Table 2***). For exploratory outcomes, *P*-values for differences between the two groups in changes from baseline to each evaluation point are also provided in ***Table 2***. No significant differences were observed between the two groups at any evaluation point for ALT, total cholesterol, triglyceride, blood urea nitrogen, serum creatinine, uric acid, or potassium.

#### Major adverse cardiovascular and cerebrovascular events (MACCEs)

***Table 3*** presents the rates of MACCEs in both groups. Overall, 6.35% of patients in the CDDP group and 5.79% in the placebo group experienced MACCEs (log-rank *P* = 0.822).

**Table 3:** Major adverse cardiovascular and cerebrovascular events (MACCEs) of patients receiving Compound Danshen Dripping Pills (CDDP) therapy or placebo

Variables	Group CDDP^a^ (*n* = 126)	Group placebo^a^ (*n* = 121)	χ^2b^	*P*-value
MACCEs [*n* (%)]	8 (6.35)	7 (5.79)	0.051	0.822
Cardiac death	1 (0.79)	2 (1.65)		
Readmission for heart failure	1 (0.79)	0		
Recurrent angina pectoris	4 (3.17)	5 (4.13)		
Stroke	1 (0.79)	0		
Unplanned recurrent revascularization	1 (0.79)	0		
^a^Eligible patients were randomized into the CDDP and placebo groups.^b^The log-rank test was used to compare the incidences of MACCEs.				

#### Arrhythmias

***Table 4*** presents the rates of arrhythmias for both groups. In the CDDP and placebo groups, 10.32% and 5.79% of patients experienced ventricular arrhythmia, respectively, and 0% and 3.81% experienced intraventricular block, respectively, which were closely related to acute anterior wall myocardial infarction. There were no significant differences in the incidence of arrhythmias between the two groups (*P* = 0.108).

**Table 4:** Arrhythmias of patients receiving Compound Danshen Dripping Pills (CDDP) therapy or placebo

Variables	Group CDDP^a^ (*n*=126)	Group placebo^a^ (*n*=121)	*χ* ^2b^	*P*-value
Arrhythmias [*n* (%)]	39 (30.95)	40 (33.06)	6.079	0.108
Ventricular arrhythmia	13 (10.32)	7 (5.79)		
Ventricular premature beat	10 (7.94)	2 (1.65)		
Non-sustained ventricular tachycardia	3 (2.38)	4 (3.31)		
Ventricular fibrillation	0	1 (0.83)		
Intraventricular block	0	4 (3.31)		
Other kinds of arrhythmias	26 (20.63)	29 (23.97)		
No arrhythmia [*n* (%)]	87 (69.05)	81 (66.94)		
^a^Eligible patients were randomized into the CDDP and placebo groups.^b^The Chi-square (*χ*^2^) test was used to compare the incidences of arrhythmias.				

### Adverse events

A total of 126 patients in the CDDP group and 121 in the placebo group were included in the safety set analysis (***Table 5***). The total number of adverse events was 46 in the CDDP group versus 36 in the placebo group (*P* = 0.260); the total number of serious adverse events was six in the CDDP group versus four in the placebo group (*P* = 0.562). No patient experienced more than one adverse event. There were 11 adverse events related to the study drugs in the CDDP group and five in the placebo group. No serious adverse events related to the study drugs were reported. The analysis of drug-related adverse events and withdrawals showed no differences between the groups (both *P* > 0.05).

**Table 5:** Summary of adverse events

Variables	Group CDDP^a^ (*n* = 126)	Group placebo^a^ (*n* = 121)	*χ* ^2b^	*P*-value
AEs [*n* (%)]^c^	46 (36.51)	36 (29.75)	1.270	0.260
Stomach discomfort and nausea (*n*)	4	2		
Itching (*n*)	1	1		
Liver function impairment (*n*)	18	21		
Renal function impairment (*n*)	6	2		
Uric acid elevation (*n*)	11	6		
SAEs [*n* (%)]	6 (4.76%)	4 (3.31%)	0.066	0.797
Death (*n*)	1	2		
Hospitalization (*n*)	5	2		
Worsening heart failure (*n*)	3	2		
Stroke (*n*)	1	0		
Interventional treatment (*n*)	1	0		
AEs related to study drugs [*n* (%)]	11 (8.73)	5 (4.13)	2.154	0.142
Withdrew due to study drugs [*n* (%)]	1 (0.79)	1 (0.83)	–	1.000
^a^Eligible patients were randomized into the CDDP and placebo groups.^b^The Chi-square (*χ*^2^) test (AEs and AEs related to study drugs), continuity correction *χ*^2^ test (SAEs), and Fisher's exact test (withdraw due to study drugs) were used to compare the incidences of adverse events.^c^The analysis included all patients who received the study medication before the events occurred. None of the patients reported more than one event, and all of the adverse events, except SAE, were assessed as grade 1 according to the CTCAE version 4.0.Abbreviations: CDDP, Compound Danshen Dripping Pills; AEs, adverse events; SAEs, serious adverse events.				

## Discussion

In this randomized clinical trial in Chinese patients with AAMI, an adjunctive therapy with CDDP alongside guideline-directed treatments significantly improved LVEF, with benefits evident at 24 weeks and persisting through the 48-week follow-up. No significant differences in the incidence of adverse events were observed between the CDDP and placebo groups. These results suggest that CDDP has the potential to improve cardiac contractility without causing significant adverse reactions. However, because of the limited sample size, future studies with larger sample sizes and longer follow-up durations are needed to validate these findings.

CDDP is a widely used traditional Chinese medicine for treating angina pectoris and diabetic retinopathy, but it is not indicated for myocardial infarction-induced cardiac dysfunction. Several studies conducted in China have investigated the effect of CDDP on ventricular remodeling following AMI^[19–21]^, and generally found a beneficial effect on NT-proBNP levels, LVEF, and ventricular remodeling in patients with AMI undergoing PPCI, compared with control groups. The MICD-STEMI PCI study^[19]^ demonstrated that CDDP reduced the risk of ventricular aneurysm formation in patients with AMI, and Shi *et al*^[21]^ found that CDDP might inhibit LV remodeling, possibly by reducing serum interleukin-6 and interleukin-8 levels. However, the relatively small sample sizes in these previous studies may have limited the generalizability of their findings.

The current study was a randomized, double-blind, placebo-controlled, multicenter clinical trial designed to evaluate the efficacy and safety of CDDP as an adjunctive treatment to guideline-directed therapies in Chinese patients with AAMI. With the expansion of the sample size, and given the strong correlation between anterior myocardial infarction and an increased risk of heart failure, the current study focused specifically on patients experiencing their first episode of AAMI. This approach allowed for a more precise investigation of the effect of ventricular remodeling on cardiac function, while minimizing the confounding effects of pre-existing myocardial infarction scar tissue on the study outcomes. Our results demonstrated that CDDP improved cardiac function at both the 24- and 48-week follow-up assessments.

NT-proBNP is a biologically inactive fragment of brain natriuretic peptide released primarily by the ventricles in response to myocardial wall stress and increased intravascular volume. It is a well-established biomarker for the diagnosis and prognosis of heart failure. Elevated NT-proBNP levels are strongly associated with the increased morbidity and mortality in heart failure patients, particularly when levels rise acutely^[22–24]^. The results of the current study indicated that CDDP did not significantly reduce NT-proBNP levels or lower the risk of MACCEs and arrhythmias, compared with the placebo. Additionally, there were no significant differences in the echocardiographic indices representing ventricular remodeling, including LVDd and LVDs, between the CDDP and placebo groups. These findings suggest that CDDP may improve cardiac function through mechanisms independent of its effects on sodium excretion and facilitating micturition, while also demonstrating a reduced likelihood of causing ventricular remodeling. Improving energy metabolism and maintaining normal glucose levels after acute myocardial ischemia to enhance myocardial contractility may explain the improvement in LVEF at 24 and 48 weeks^[10]^. Furthermore, no serious adverse events were related to CDDP, because the incidence of adverse events did not differ significantly between the two groups.

Cardiac function post-AAMI was generally well-preserved, with baseline LVEF values exceeding 50% in both groups. Several factors may contribute to this observation. First, early reperfusion of infarct-related coronary arteries through PCI salvages ischemic myocardium, reduces the infarct size, and minimizes myocardial cell loss, playing a crucial role in preventing or delaying heart failure. Second, therapies aimed at preventing cardiac remodeling, such as β-blockers and ACEIs or ARBs used in guideline-directed medical therapy, contribute to delaying the early onset of heart failure. Additionally, the symptom-to-door time for AAMI patients with LV systolic dysfunction (LVEF < 50%) often exceeds 12 h because of the delayed pain response in the elderly and prolonged transport time, which limits the recruitment of patients with lower baseline LVEF.

However, the current trial had some limitations. Approximately 21% of the enrolled patients, which exceeded the 10% attrition rate assumed during sample size estimation, were ineligible for primary outcome analysis because of the challenges in the follow-up arising from the coronavirus disease 2019 (COVID-19) pandemic and other issues, such as the inconvenient transportation for patients living in remote areas. Ultimately, the statistical power was reduced to 83% from the 90% set at the time of the study design, but this remains acceptable in clinical trials. In addition, multiple imputation analysis yielded results consistent with the complete-case analysis.

The current trial enrolled 247 patients and comprised a 24-week treatment period as well as a 48-week follow-up period; a larger, randomized, controlled study with a longer medication duration and a larger patient population with worse cardiac function is needed to validate our results and to investigate the underlying mechanisms of the improvement in cardiac function by CDDP in AAMI.

In conclusion, on a background of guideline-directed medical therapy, CDDP, a traditional Chinese medicine, significantly improved both 24- and 48-week echocardiographic outcomes reflecting cardiac ejection fraction without causing severe adverse events. Future research is warranted to elucidate the underlying mechanisms of action of CDDP in this context and to confirm our findings in larger and more diverse patient populations with a longer follow-up time.

## SUPPLEMENTARY DATA

Supplementary data to this article can be found online.
