# Supplementary material for: Effect of Compound Danshen Dripping Pills on cardiac function after acute anterior ST-segment elevation myocardial infarction: A randomized trial
Source: J Biomed Res. 2025 Jan 10;39(4):407–16. doi: 10.7555/JBR.38.20240325 (PMC12329410; doi:10.7555/JBR.38.20240325)
Supplement: Supplementary file 1 — Supplementary data to this article can be found online. [file jbr-39-4-407-Supplementary.pdf]

Available online at www.jbr-pub.org.cn

Open Access at PubMed Central

Journal of Biomedical Research, 2025 39(4): 1–1

Supplementary Data

JBR

Effect of Compound Danshen Dripping Pills on cardiac function after acute anterior ST-segment elevation myocardial infarction: A randomized trial

Bo Deng[1,△], Sibo Wang[1,△], Yujie Wu[1,△], Qiming Wang[1], Rui Qiao[2], Xiwen Zhang[3], Yuan Lu[4], Li Wang[5], Shunzhong Gu[6], Yuqing Zhang[7], Kaiqiao Li[8], Zongliang Yu[9], Lixing Wu[10], Shengbiao Zhao[11], Shuanglin Zhou[11], Yang Yang[1,✉], Liansheng Wang[1,✉]

[1]*Department of Cardiology, the First Affiliated Hospital of Nanjing Medical University, Nanjing, Jiangsu 210029, China;*
[2]*Department of Cardiology, Anqing Municipal Hospital Affiliated to Anhui Medical University, Anqing, Anhui 246003, China;*
[3]*Department of Cardiology, Huai'an First People's Hospital, Huai'an, Jiangsu 223300, China;*
[4]*Department of Cardiology, the Affiliated Hospital of Xuzhou Medical University, Xuzhou, Jiangsu 221006, China;*
[5]*Department of Cardiology, Pukou Branch of Jiangsu People's Hospital, Nanjing, Jiangsu 211899, China;*
[6]*Department of Cardiology, Hai'an People's Hospital, Nantong, Jiangsu 226699, China;*
[7]*Department of Cardiology, the Affiliated Jiangning Hospital of Nanjing Medical University, Nanjing, Jiangsu 211199, China;*
[8]*Department of Cardiology, Qixia District Hospital of Nanjing City, Nanjing, Jiangsu 210046, China;*
[9]*Department of Cardiology, the First People's Hospital of Kunshan Affiliated to Jiangsu University, Kunshan, Jiangsu 215300, China;*
[10]*Department of Cardiology, Nanjing Lishui District Hospital of Traditional Chinese Medicine, Nanjing, Jiangsu 211299, China;*
[11]*Department of Cardiology, Nanjing Meishan Hospital, Nanjing, Jiangsu 210039, China.*

Supplementary Table 1 **LVEF levels from baseline to four, 24, and 48 weeks after PCI (complete**

Time points	Group CDDP[a]	Group placebo[a]	Difference (95% CI)[b]	*P*-value
T_0	1.90±7.52[c]	0.67±6.77[c]	1.37 (−0.64, 3.39)	0.181
T_1	3.49±7.14	−0.03±7.90	3.49 (1.35, 5.62)	0.001
T_2	3.63±7.43	−1.13±8.45	4.71 (2.44, 6.98)	<0.001

[a]Eligible patients were randomized into the CDDP and placebo groups.
[b]The differences from four, 24, and 48 weeks to baseline between the two treatment groups are based on the difference of least square means of linear mixed effects models for repeated measures.
[c]The mean changes of LVEF in both groups are calculated based on the number of patients having actual LVEF values at the baseline or the 24- and 48-week visits.
Abbreviations: T_0, four weeks after PCI; T_1, 24 weeks after PCI; T_2, 48 weeks after PCI.

△These authors contributed equally to this work.

✉Corresponding authors: Liansheng Wang and Yang Yang, Department of Cardiology, the First Affiliated Hospital of Nanjing Medical University, 300 Guangzhou Road, Nanjing, Jiangsu 210029, China. E-mails: drlswang@njmu.edu.cn (Wang) and yangyang@jsph.org.cn (Yang).

Received: 04 October 2024; Revised: 30 December 2024; Accepted: 03 January 2025; Published online: 10 January 2025

CLC number: R542.2, Document code: A

The authors reported no conflict of interests.

This is an open access article under the Creative Commons Attribution (CC BY 4.0) license, which permits others to distribute, remix, adapt and build upon this work, for commercial use, provided the original work is properly cited.

© 2025 by Journal of Biomedical Research.

https://doi.org/10.7555/JBR.38.20240325
